# Strengthening the European Semester to Achieve Economies of Wellbeing

**DOI:** 10.3390/ijerph21050634

**Published:** 2024-05-16

**Authors:** Ingrid Stegeman, Vania Putatti, Alba Godfrey, Caroline Costongs

**Affiliations:** EuroHealthNet, 1000 Brussels, Belgium; v.putatti@eurohealthnet.eu (V.P.); a.godfrey@eurohealthnet.eu (A.G.); c.costongs@eurohealthnet.eu (C.C.)

**Keywords:** economy of wellbeing, European Semester process, integrated governance, European Union, sustainable development

## Abstract

The environmental crisis, growing levels of social inequalities and rising levels of noncommunicable diseases are all symptoms of economic systems that are failing to generate wellbeing. There is increasing support for the notion that addressing these crises requires shifting the focus from economic growth to a broader range of measures that reflect wellbeing, through more comprehensive, consistent and integrated policy approaches to deliver this. In 2019, the EU Finnish Council Presidency Council Conclusions called amongst other things for the development of a new long-term, post-2020 strategy to provide the framework for horizontal assessment and cross-sectoral collaboration, in particular through the European Semester process. This article contextualises this call and explores its follow-up. It draws from key policy documents to explore what Economies of Wellbeing are, why and how the concept has emerged and how they can be put in place. It then explores to what extent this concept is being applied at the EU level, by tracking changes in some of the EU’s key policies and strategies over the past 10 years and in the Semester process, as a mechanism to implement them. It concludes that while progress towards more comprehensive, consistent and integrated policy approaches has been made in the context of the Annual Sustainable Growth Strategy, underpinning the Semester processes, it is limited by the continuing emphasis on economic, over other policy, areas. It also argues that the process needs to be broadened even further, to include other dimensions of wellbeing, which intersect with the economy and impact wellbeing. To strengthen the European Semester process to achieve Economies of Wellbeing, it should be put at the service of an even more consistent and comprehensive EU Strategy that enables policy sectors to deliver wellbeing objectives in a more integrated and coordinated manner. This paper ends with recommendations for action.

## 1. Introduction

Article 3 of the Treaty of the European Union sets out that the aims of the Union are peace, democracy and the wellbeing of its people. The economy is critical to achieve these overarching objectives. It is perhaps the most important underlying determinant of health, affecting other key determinants, like good-quality environments, adequate income, housing, a sense of safety, security and belonging, purpose and participation [1] While the economy should be in the function of wellbeing, economic growth has become the objective, with too little regard for how and if it is contributing to this goal.

The Economy of Wellbeing is a policy orientation and governance approach that aims to put the concept of wellbeing back at the centre of economic systems, to reorient their performance towards this goal. The concept has gained prominence, also at the international policy level, as it is becoming increasingly clear that the environmental crisis, growing levels of social inequalities and rising levels of noncommunicable diseases are all symptoms of an economic system that is failing to generate wellbeing.

Part 1 of this paper will set out the evolution of the Economy of Wellbeing, differences in conceptualisation and how it can be implemented. While the use of terms like the Economy of Wellbeing or Wellbeing Economy reflects different views on the extent of systemic change needed to achieve economies that generate more wellbeing, the terms will, in this paper, be used interchangeably. Both terms reflect a pressing need to transition to economic systems that perform better in terms of improving people’s lives, through better collaboration across sectors to achieve this. Part 2 sets out the possibilities and challenges of integrating this concept at the European level and focuses on the European Semester as a governance mechanism that can be used to mainstream this process across the EU. It will explore the process’s evolution from a mainly economic coordination mechanism between EU Member States to one that takes into consideration a much broader range of factors impacting and impacted by Member States’ economies. The final part will explore how this evolution represents a move towards an Economy of Wellbeing and the factors inhibiting this. It will discuss the need to put the EU Semester process at the service of a more comprehensive and coherent and bolder EU-level strategy and framework that places the wellbeing of people and the planet at its centre. It will also set out recommendations for reform and progress, to move closer to these objectives. 

This article builds on EuroHealthNet’s work on the European Semester process, over the past ten years, where we explored country-specific recommendations in relation to health inequalities [2]. It also builds on our work on Wellbeing Economies, as an approach to tackle the underlying determinants of health and “get things right the first time” around problems like mental and physical ill health, homelessness and crime [3]. This paper aims to improve the understanding of these two complex topics and how they intersect. As a network of public bodies operating in the EU policy arena, we based our analyses on policy documents from the EC, OECD, WHO and other organisations and think tanks like the Wellbeing Economy Coalition, and ZOE Institute of Future-fit Economies, supported with further desk research. We also drew on the work of seminal economists and thinkers in the field, like Joseph Stiglitz, Marianne Mazzucato, Katherine Trebeck and Kate Raworth.

## 2. What Is an Economy of Wellbeing and How Can It Be Shaped? An Overview

The need to shift to economies that deliver more wellbeing has been gaining attention and adherence in recent years in the EU and its Member States and across the world. This is a response to the growing awareness of how economic growth can come at the cost of the environment, equity, public health and the very wellbeing it is meant to generate. More balanced approaches are needed, based on the assumption that economic prosperity, environmental protection and equitable wellbeing can and should be interconnected elements of a more holistic agenda [4]. The concept of wellbeing can therefore act as an increasingly relevant compass for policy to ensure economies become better at delivering this for people and the planet; this also entails improving governing tools and mechanisms to achieve this objective.

### 2.1. The Predominance of GDP and Its Consequences

In Europe and across the world, economic growth is normally measured through Gross Domestic Product (GDP), which is the monetary value of all finished goods and services made within a country during a specific period [5]. Simon Kuznets, who developed the measure in 1934, warned, however, that GDP should not be equated with economic or social wellbeing. The figure included many goods and services that can be considered harmful (e.g., armaments or prostitution) or useless (financial speculation) and excluded many essential ones that are free (such as caregiving by homemakers) [6,7]. In addition, GDP does not reflect how goods are distributed in society nor does it measure health, education, the equality of opportunity, the state of the environment or many other indicators of the quality of life or the sustainability of the economy. Cleaning up the mess of pollution or services that address the consequences of ill health, for example, will increase GDP, while cleaner, healthier environments will not necessarily do so. As the economist Mariana Mazzucato sets out, anything that fetches a price is GDP, even if it does not create but in fact extracts value [7].

Nevertheless, GDP became hegemonic across the globe, as a metric released with high frequency, computed according to internationally agreed standards, making it ideal for cross-country comparisons. There has been a growing faith since the 1970s in the efficiency of markets to deliver growth and prosperity, and GDP became “both the numerator and denominator” of development [8]. Good economic policy became that which increases this single indicator the most, and required increasing consumption, to improve living standards of all and remedy some of society’s most pressing challenges [1,9].

The multidimensional and interconnected crises that societies across the globe are facing, like environmental degradation and climate change, rising levels of social and health inequalities and growing public health challenges, reflect how current economic systems are failing to generate health and wellbeing. Even when economies are flourishing, many people are not [9]. The current economic system is the major driver of climate change and biodiversity loss [10,11,12]. As a result, planetary systems are changing and becoming less predictable and thereby less capable of sustaining human life. While the relationship between the economy, the environment and health is not straightforward, there is strong evidence that exposure to the natural world is essential for good health. People and places that are already less and the least well-off are paying the highest price for environmental degradation [12,13].

The financial crisis in 2008 exposed the inherent lack of fairness in current economic systems, as financial and corporate interests that generated the crises were protected, at the expense of the more and most vulnerable members of society [11,12]. More recently, the COVID-19 pandemic raised the general public’s awareness of how these dynamics are at play, within Europe and beyond, through vaccine inequality. Economic shocks from the pandemic impacted those who were already disadvantaged, compounding their difficulties. EU institutions and governments learned the lesson to invest in human and social capital, to enable people and the economy to withstand economic shocks [14,15] Nevertheless, wealth inequalities continue to grow within and between many EU Member States, reducing social cohesion and undermining wellbeing [16,17,18].

Rising rates of noncommunicable diseases (NCDs) across Europe and the world also reflect how current economic models are failing to generate health and wellbeing. New technologies can enhance “productivity” but encourage sedentary lifestyles, longer working hours, new forms of addictions and more waste. This eats into time spent caring for children, parents and others, which in economic terms, has very little or no value. It can also affect the amount or quality of leisure time available to restore health and wellbeing through non-productive pursuits. Studies demonstrate that psychological distress has increased exponentially, especially at times of accelerated economic growth, and aim to explain why societies are increasingly plagued by anxiety, depression, narcissism, reduction in empathy and other mental disorders [19]. Loneliness is also widespread among Europeans: in 2018, 75 million people in Europe felt socially isolated [20].

While people across Europe may live longer than ever before, the extra years are not spent in good health, with the advances that have been made in life expectancy over the past century stagnating and even declining in some countries. There are plenty of medical innovations and solutions which, for example, treat noncommunicable diseases like obesity, heart disease and respiratory diseases and “boost GDP” but do not address the underlying causes of ill health, such as pollution or unsustainable food systems or the lack of a sense of purpose, worth and belonging, nor do they contribute to collective wellbeing in the long run [21] The costs of not protecting the environment and health are likely to be much greater than the investments and measures required to conserve these [22,23].

### 2.2. Growing Support for Moving “Beyond GDP” and the Wellbeing Economy

Economists like Mazzucato and Raworth point out that economics is a social science, and economies are not things in themselves; they are shaped by societies and outcomes of multi-agent processes in a specific context and are deeply embedded in social and political institutions [7,13]. Discussions on the need, and the best approaches to reshape economies, to achieve more inclusive, just and sustainable societies have taken place for decades. A common approach has been to identify new ways to measure economic and social progress, given that “what we measure determines what we do” [24]. The aim of such approaches is to shift the emphasis from economic growth, as the measure of economic and social progress, to other indicators, “beyond growth”, and/or to change the way in which we measure growth. The Human Development Index was, for example, developed in 1990 to emphasise that people and their capabilities should be the ultimate criteria for assessing the development of a country, not economic growth alone [25]. The Genuine Progress Index was developed in 2006 as a metric that differentiate between economic activity which diminishes both natural and social capital and activity that enhances these capitals [26]. Terzi (2021) posits that such indexes were not widely taken up, for practical and political reasons [9].

In 2008, France’s president Nicolas Sarkozy realised that any politician who single-mindedly sought to push GDP to the neglect of other indicators of the quality of life risked losing the confidence of the public. He set up an international Commission on the Measurement of Economic Performance and Social Progress, headed by economist Joseph E Stiglitz [6]. Their work was taken up by the OECD and lay the foundation for the “Better Life Index” that was published in 2011, to account for the complexity of economic and social progress [27]. The index ranks 11 dimensions equally, to compare the status and progress of economies. Aligning with this, the European Commission produced a Communication *‘GDP and beyond: Measuring progress in a changing world’* in 2009 and developed several new quality of life indicators, leading to new surveys and regular reporting on these data.

There is, therefore, no shortage of initiatives or indicators to reconceptualise economic prosperity and social progress. In 2019, the OECD listed around 500 initiatives that are being used by researchers, NGOs, countries and international organisations to measure development beyond GDP [8]. The challenge lies in mainstreaming these initiatives, at a large enough scale and in a consistent and coherent enough way to shift the focus from growth to a broader set of measures that reflect how economies are performing in terms of what matters the most for addressing the needs of people and the planet. Many of the new indicators, like the EU’s quality of life indicators, remain confined to their respective policy areas, do not obtain much media attention and are not robust enough to be used alongside GDP, as a reflection of economic performance [24] (see also Section 2.4).

The United Nations’ 2030 Agenda for Sustainable Development (2015) which considers the economic, social and environmental dimensions of sustainable development represents perhaps the most consensual view at the global level of what constitutes a prosperous society. While all UN Member States have agreed to try to achieve the goals by 2030, its breadth (17 goals and 169 targets) is considered by many to limit their efficacy in steering policy in a specific direction [6,19,24].

**Figure 1:**
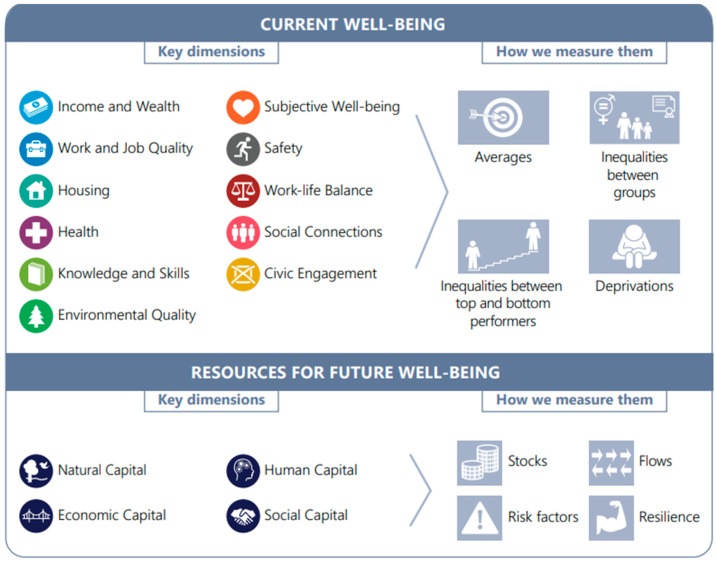
OECD Wellbeing Framework: How’s Life: Measuring Wellbeing (2020) [29].

Building on these initiatives, and to maintain the momentum of efforts to reshape economies, to achieve more inclusive, just and sustainable societies, the OECD first used the term “Economy of Wellbeing” in a Working Paper on the topic, published in 2019 [28]. It called wellbeing an “increasingly relevant compass for policy” and defined the Wellbeing Economy as one that achieves the following: it expands the opportunities available to people for upward social mobility and for improving their lives along the dimensions that matter the most to them; ensures these opportunities translate into wellbeing outcomes for all segments of society, including those at the bottom of the distribution; reduces inequalities and fosters environmental and social sustainability. The report sets out the OECD’s “Well-Being Framework” (see Figure 1) that includes 11 dimensions which reflect extensive evidence from the fields of neuroscience, epidemiology and psychology about what constitutes a good quality of life The model also includes four kinds of capital: natural, human, social and economic, which must all be considered, to ensure that impacts on the future are recognised. 

The report sets out how policies in education and training, health, social protection and redistribution and gender equality should be considered investments that drive both wellbeing and economic growth. It calls on governments and other sectors to seek growth in ways that improve wellbeing, which can in turn generate growth, in a mutually reinforcing circle.

This concept of an Economy of Wellbeing found support in that same year during the Finnish Presidency of the Council of the European Union, which made it a major theme during their Presidency, which led to the EU Member States agreeing to EU Council Conclusions on this topic. The conclusions stress that wellbeing is “vitally important to the Union’s economic growth, productivity, long-term fiscal sustainability, and societal stability”. They call on the EU Commission and Member States to set evidence-based objectives to achieve growth in ways that enhance human and social capital and improve wellbeing and to implement efficient and equitable policy measures and structures measured through clear indicators. They invite the EU Commission and EU Member States to develop communication (policy proposal) around this topic and to develop a future EU strategy to “ensure that the Union becomes the world’s most competitive and socially inclusive, climate-neutral economy, reflecting the economy of wellbeing.” Such a strategy should stimulate “a whole of society … horizontal approach that can seize synergies across different policies and ensure that policy measures in one part of government are aligned with ambitions in another part.” The Council Conclusions also note that the European Semester, which provides a framework for the coordination of Member States’ economic policies, is an important tool for monitoring the implementation [30].

Support for the concept of Wellbeing Economies is also apparent in the growth of the Wellbeing Economy Alliance (WEAll), which was established in 2019 to take forward the visions of Katherine Trebeck, Jeremy Williams [31] and other leaders and activists in the field. WEAll is an international collaboration of more than 400 organisations and thousands of individuals who agree that “our economic system is not working fairly for everyone and that we need to put people and planet in front of profit.” WEAll therefore calls for a much more fundamental shift in economic systems and conventional approaches to governance. It defines a Wellbeing Economy as “one designed to serve people and the planet, and not the other way around”. WEAll describes itself in terms of values (Participation; Nature; Purpose; Dignity) and principles (Pre-distribution, Purpose, Prevention and People). It aims to transition to economies in which people see themselves as caretakers and creators, rather than owners and consumers. It emphasises participative approaches, subsidiarity and community action, for agency, ownership (of concepts rather than things) and belonging [32].

Another influential framework and movement in close alignment with the values and principles of Wellbeing Economies is the Doughnut Economy, named after the model used to illustrate the concept by economist Kate Raworth. She proposed the model in 2012 and later refined it in her book of the same name [13] The model depicts the “safe space for humanity” that could also be characterised as the space of wellbeing, in which the needs of all people are met in a way that does not overshoot ecological boundaries. Doughnut Economics promotes cooperative and caring human behaviour and advocates for transforming degenerative and divisive economies into regenerative and distributive ones (that applies relevant data from the EU to the model).

These ideas have not remained in the realm of theory, as the governments of Scotland, Iceland, New Zealand, Wales, Finland and Canada (Wellbeing Economy Governments WEGo) have joined forces to apply them. These governments have, through different kinds of processes, defined wellbeing goals and indicators and are using them to guide policy and budgetary decisions. WEAll was involved in the creation of WEGo, but the group now operates independently, with the support of the Scottish government [33]. Five WEGo have harnessed multidimensional wellbeing frameworks, to reshape their economic models (see examples at the end of this section.) The Doughnut Economics model is also being used by local governments across the world, who are part of the Doughnut Economics Action Lab [34].

The COVID-19 pandemic provided further impetus to the “Beyond GDP” and wellbeing movements, at the EU policy level and beyond. In 2021, for example, European heads of government signed the Porto Declaration, calling for an alternative set of indicators to measure economic, social and environmental progress, supplementing GP as a welfare measure for inclusive and sustainable growth [35]. The European Economic and Social Committee (EESC) also developed an opinion on the Wellbeing Economy [36]. In 2023, the European Parliament hosted a large conference entitled *“Beyond Growth: A Blueprint for a Social and Green Deal”* [37]. The World Health Organisation (WHO) has also been very active, since 2019, in exploring and promoting the concept of Wellbeing Economies, to promote investments in health [38,39,40,41]. Another significant development is proposals by the United Nations to change the National Statistical System to complement GDP that will be presented in 2025. The aim is to design metrics as equally clear and appealing as GDP, to change the focus of policy-making towards more sustainable, just and inclusive development [8].

### 2.3. Differences of Opinion on How to Achieve Economies That Deliver More Wellbeing

While the abovementioned initiatives and movements reflect growing acknowledgment and support for economies that generate more wellbeing, they also reflect differences in how this shift can be achieved and economies and societies reshaped. There are, on the one hand, people and organisations that maintain that economies (and societies, with economies as their central driver) should be focused on “growth” but that more scrutiny is needed around what and how this is being “grown”. Mainstream organisations that represent governments, like the OECD, EU and UN, including the WHO, subscribe to this approach, referring to “green growth” or “sustainable and inclusive growth”. They believe that it is possible to decouple GDP growth from negative environmental or social impacts and aim to achieve Economies of Wellbeing by “integrating a new, more wholistic, perspective into existing activities”, as set out in information about the Finnish Action Plan on the Economy of Wellbeing [42] (see below).

Others, on the other hand, stress the need to shift the focus away from growth altogether and to focus instead on the end goals of what matters to people and their wellbeing, as this will determine and reshape the nature of economic transactions and the market. They also point to the mounting evidence of the negative externalities of growth and the lack of empirical evidence that economic growth can be sufficiently decoupled from its environmental impacts to the scale needed to address climate breakdown [43]. Proponents of “de”- or “post”-growth acknowledge, however, that this is a long-term process, since “any vision of growth is up against formidable structural dependence”, since public services, such as social spending, including pensions, unemployment benefits, health services, education, reskilling programmes, and military services, which are needed to maintain economic and social stability, depend on economic growth [31]. While, therefore, WEAll promotes the de-prioritisation of economic growth as a policy objective, WEGo remain more focused on complementing GDP as a measure of performance with other indicators [44].

### 2.4. How to Design Economies of Wellbeing

Despite differences in views on how to achieve wellbeing societies, the entry points for change are similar. All approaches require instigating a shift from thinking about the economy in terms of “growth” to “wellbeing”, breaking through more siloed approaches to governance, and encouraging and enabling public and the private sector actors to hold multiple perspectives of objectives at once, to contribute to wellbeing.

There is no prescription for social change in highly complex, democratic societies with interconnected challenges, as this involves “a change in practices, policies, lifestyles and mental models that destabilise and phase out bad practice, while building the resilience of good practices” [20]. Numerous actors have developed useful guidance on how to generate such change, and identified a number of requisites. An important requisite for social change is for example “entrepreneurial governments” that recognise their role as going beyond facilitating markets and addressing market failures, towards a more direct pursuit of specific public goods, as set out by the work of Mariana Mazzucato [45]. Another requisite is for strong new narratives and frames around what constitutes economic prosperity and social progress, which requires close collaboration with the media and communication specialists to develop and apply these, in a consistent way. The Finnish government convened a high level international expert group that set out “priorities for promoting an Economy of Wellbeing” [46]. The ZOE institute for Future-fit Economies has also published useful guidance on how to develop the kinds of horizontal approaches and integrated policy measures and structures mentioned in the Finnish Presidency Council Conclusions [20,47,48]. WEAll has published guidance on how governments and other social actors can achieve Wellbeing Economies [49]. Drawing from the extensive literature in the field, the guidance sets out how this requires, first and foremost, identifying the policy priorities that reflect what matters most to a specific constituency. As discussed, expressions of this tend to converge around material security, physical security, political voice and social connection. Nevertheless, these priorities may be articulated in different ways by different groups of people, depending on the cultural, geographical or administrative level and context.
In December 2013, the parties in a coalition government in **Germany** indicated that they wanted to align their policies more closely with the values and hopes of German citizens. They therefore conducted a **national Wellbeing dialogue process** that led to 203 national dialogue events across the country, from April to October 2015. People who were not able to take part in person could provide input through a website or by post. A total of 15,750 people took part in the dialogues. An independent team of scientists analysed the responses, which led to a government strategy on **“Wellbeing in Germany-what matters to us”**. These priorities were then organised into three broad categories with 12 dimensions: (1) ‘Our life’, which describes five dimensions: health, work, education, income and the time we have available. (2) ‘Our surroundings’, which covers three dimensions of lives: where we live, infrastructure and mobility in our cities and rural areas, security and social cohesion. (3) ‘Our country’, which forms the national and international framework and relates to the economy and environment, being able to live in freedom and equality and the concerns of citizens about peace and Germany’s responsibilities in the world [49].

Governments must then ensure their policy priorities are consistent, coherent, well integrated and coordinated. Table 1 provides useful definitions of these terms, set out by the ZOE Institute of Future-Fit Societies [47].

Ensuring policy coherence, consistency and integration is best achieved through the development of an overarching strategy, which sets out common priorities/objectives and associated targets and indicators, that can be used to guide policy implementation and assess outcomes. Policies (e.g., legislative acts, regulations, incentives, disincentives, information campaigns, public provisioning) must then be developed or reformed to take forward the different objectives of the strategy. Tools and mechanisms must also be developed that encourage and enable policy-makers and businesses to assess the impacts not just on the policy priorities that are of the most relevance and interest to them but also on other policy priorities, so that trade-offs can be established, synergies made and tensions addressed.

The guidance on how to achieve wellbeing economies, through more holistic approaches to policy-making, stresses the importance of engaging a wide range of stakeholders during all phases of the policy cycle (development, implementation and evaluation). This helps to ensure that certain dimensions or population groups are not overlooked or subordinated and to ensure that different stakeholders, not least those in economic and finance sectors, recognise the value and co-benefits of adopting a more holistic perspective. Stakeholder engagement in all steps also helps to build a common understanding and language around policy priorities, to ensure resonance. This is needed to break through siloed approaches and to obtain buy-in around new or improved structures and mechanisms to facilitate better integrated, coordinated, holistic approaches. Such buy-in is also needed to secure investments in platforms to share relevant data across departments and organisations and to remove unnecessary legislative, financial or administrative barriers to cross-sectoral collaboration. 

While the need to address specific policy priorities like the environmental crisis is urgent, imposing decisions too quickly without adequate stakeholder buy-in can backfire if opposition emerges and they are overturned in new election cycles. A case in point is plans and regulations introduced under the European Green Deal to “fast track” efforts to achieve environmental policies. The Green Deal now faces considerable opposition, in the face of EU-level elections. As Barth et al. set out, a less quick and often overlooked but more sustainable way to shift societies towards greater wellbeing is through governance mechanisms and tools, including metrics that embed a more balanced consideration of wellbeing dimensions into governance processes [20]. These metrics should, e.g., be applied to targets, and used in reporting and monitoring procedures as well as ex-post and ex-ante impact assessment tools, and used to trigger enforcement mechanisms. These metrics can also be used to determine budgeting priorities and to condition the use of public funds, to ensure that they are channelled to achieve the relevant priorities. Instead of, for example, automatically using GDP as a measure of funding needs in specific localities, measures that reflect inequity, or pollution levels, could instead be used to target other, more specific wellbeing challenges that areas are facing. Embedding new indicators that reflect different dimensions of wellbeing into governance tools will strengthen demand for the new indicators and data and improve their quality, thereby generating positive feedback loops/dynamics in their use, to complement or displace GDP and mainstream wellbeing priorities.
The **National Statistical Institute in Italy has developed a Measure of Equitable and Sustainable Wellbeing (BES) that includes 12 indicators** like S80/20, greenhouse gas emissions and disposable income. These indicators have, since 2018, been **used to forecast and discuss the budget law** (‘Legge di Bilancio’). One of the criteria for selection was that they had to be forecastable over a short (three-year) horizon, and for this reason, subjective wellbeing was excluded. The Italian Ministry of Finance has indicated its intention to monitor the EU Recovery and Resilience Plan based not only on the basis of macroeconomic and employment indicators but also through the BES scoreboard [9].

It is, however, very difficult to find metrics that are conceptually clear enough, and for which data can be collected in a timely enough manner, to be useful for such policy purposes. A reason GDP has gained its dominance as a measure of economic prosperity and social progress is because it is easy for everyone to understand (up is good and down is bad) and because it has been refined through widespread and consistent use, to generate data that are easily available. It will not be easy to cultivate the use of other metrics that can be used alongside GDP to reflect other wellbeing-related outcomes. Ensuring the consistent use of the same indicators, within and between countries, is also important, since comparison is an important driver of behaviour change. The UN’s proposals of indicators that can be used by National Statistical Systems, to complement GDP, could be significant for efforts toward mainstream indicators “beyond GDP” [8]. This points, however, to tension between the need to engage people in the process of identifying priorities and metrics related to wellbeing, and the need to identify metrics that will be widely and consistently used within and between countries, to reinforce familiarity and use and to refine them.

Achieving the conceptual shift towards greater wellbeing for people and the planet entails a long-term, multi-pronged process. While the path towards such change can seem daunting, WEGo like Wales, Iceland and Finland have embarked on the journey to transition to economic systems that generate more wellbeing.
Wales launched the **“Wellbeing of Future Generations Act”** in 2015 in the aim of improving decision-making towards the achievement of seven wellbeing goals (prosperous, more equal, globally responsible, resilient, healthier, cohesive communities, vibrant culture and thriving Welsh language). The Act, embedded in the Welsh constitution, requires all public bodies to consider the long-term impact of their actions, collaborate across sectors and involve communities. It has succeeded in changing the way Wales evaluates progress and success, which are now based on wellbeing rather than GDP. A Future Generations Commissioner helps to monitor the extent to which the wellbeing objectives are met. The 2020 Future Generations Report includes an overview of progress towards the seven wellbeing goals by public bodies [49].

**Iceland has adopted a system-wide approach to wellbeing and introduced six wellbeing priorities** (mental health, secure housing, better work/life balance, zero carbon emissions, innovation, growth and better communication with the public). These were co-developed with stakeholders and serve to guide Iceland’s Five Year Fiscal Strategic Plan. Progress is assessed through public health and wellbeing surveys, as well as a new wellbeing economy indicator system introduced in 2019. Iceland’s prioritisation of wellbeing, exemplified in taxation and labour market policies that invest in and protect the most vulnerable households, was instrumental in helping the country recover from the 2008 recession [50].

Finland’s national vision, **The Finland we want**, brings together existing sector-based long-term strategies within one overarching framework, with a common timeline for 2050 [51].The focus of the **Finnish Action Plan for the Economy of Wellbeing (2023–2025)** is to develop a common definition of social sustainability and indicators that reflect this. It will also develop impact assessment tools, which integrate this information. The aim is to ensure that the indicators and tools describing wellbeing are increasingly used in decision-making along with information describing economic and environmental sustainability, at national, regional and local decision-making levels [52]

## 3. The ‘Broadening and Softening’ of the EU Semester Process

### 3.1. Wellbeing as the Overarching Aim of the European Union

As reflected above, transitioning to economies that deliver more wellbeing calls for a whole-of-society approach, with vertical and horizontal integration and collaboration, to ensure a more balanced consideration for all dimensions of wellbeing, not just economic growth. As a supranational body that has a considerable influence over its Member State economies, the EU could play a significant role in encouraging them to transition to economies that generate more wellbeing. There is, on the one hand, good potential for this, given that the EU’s overarching aim is wellbeing. According to Article 3 of the Treaty on the European Union (TEU), the “Union’s aim is to promote peace, its values (of, e.g., freedom, democracy, equality and respect for human rights) and the well-being of its peoples”. The TEU also states that these aims will be achieved through the “sustainable development of Europe based on balanced economic growth and price stability, a highly competitive social market economy, aiming at full employment and social progress, and a high level of protection and improvement of the quality of the environment” [53]. Various other provisions in the EU Treaties, including Article 9, which was included in the Lisbon Treaty (TFEU) as a “horizontal social clause”, set out objectives that are directly or closely related to protecting and promoting public health and wellbeing [54].

The EU’s role in stimulating the transition in Member States to economies that generate more wellbeing is, on the other hand, limited by the fact that a prioritisation of economic areas, over other areas, and a focus on “GDP growth” have been locked into its institutions, in ways that are very hard to undo. The EU’s founding fathers held the belief, still predominant today, that the main way to achieve these aims was through economic growth, to satisfy citizens’ material needs. The main function of the EU level of governance was therefore to establish and enforce the rules for a common market. EU institutions were entrusted with tasks that could be solved on the basis of economic rationality, while the political sensitive decisions of redistribution, requiring broad social consensus, were left to the national governments [55]. As a result, EU institutions can directly develop and enforce laws in areas like a customs union, competition rules, a monetary policy or trade, while they share power with Member States in areas like the single market, employment, social affairs and the environment. The EU institutions only provide support to Member States in policy areas like public health, education and training through guidance, exchange and funding mechanisms, to encourage “upward convergence” [56].

The fact that the EU’s main objective has been to deliver economic growth, the stronger powers bestowed upon it in these areas and that its social market economy model has been very successful, by global standards, in generating prosperity in inclusive ways, makes it difficult to argue the need for change [57,58]. Nevertheless, the interconnected environmental, health and social crises that are threatening democracies can be traced to economic models driven by growth, with too little scrutiny for the nature and the direction of that growth. In their conclusions, the members of the High-Level Group on Wellbeing convened by the Finnish noted that “we know the limits of natural and manmade capital, while we do not yet know the limits of human and social capital” [46]. It is notable, however, that these are the areas in which the EU has the least power to legislate.

Despite these constraints, there are positive signs that the EU is encouraging Member States to reform their economies into one “that works for people and the planet.” The next section will trace developments that have moved it in this direction and towards greater policy consistency and coherence. It will focus on developments in the context of the European Semester process, as the EU’s main policy mechanism for socioeconomic policy coordination. 

### 3.2. The European Semester Process

The European Semester process (henceforth also Semester) aims to achieve greater coherence and convergence across Member State actions. Its purpose is to steer policies of EU Member States, to ensure their economic sustainability and that of the EU. It brings together a wide range of EU governance instruments with different legal bases and sanctioning authority and has given the EU Institutions greater influence in scrutinising and guiding economic and fiscal policies but increasingly also others, especially within the euro area [59,60]. The process is a “highly technical, evidence-based” one that initially focused almost exclusively on monetary and economic policy, which many consider to have harmed rather than helped the performance of many EU economies [61]. As will be set out below, it has been reformed into a tool that now aims to foster a more cohesive socioeconomic environment, encourage sustainable and environmentally friendly growth and enhance economic performance across the EU Member States [62,63].

### 3.3. The Emphasis on Fiscal and Macroeconomic Considerations 

The European Semester process was first initiated in 2010, as an integral component of a comprehensive set of measures to strengthen EU economic governance in the aftermath of the financial and sovereign debt crises. The crises revealed how interdependent European economies are and the necessity of strong economic and fiscal coordination in the EU, to ensure the existence and respect for rules and restore trust and confidence in markets. The EU Member States therefore set up an annual policy coordination cycle, to review developments and progress in various thematic areas using jointly formulated indicators and benchmarks in addition to financial support through various programmes. While the focus was primarily on Member States’ economic and fiscal policies, it was also intended to coordinate their policies towards the achievement of the overarching Europe 2020 Strategy, which included social and environmental objectives.

The annual cycle was and remains as follows: the European Commission (EC) outlines a strategic overview, initially called an “Annual Growth Survey”, that sets out annual policy priorities and common guidelines for Member States. The EC then works with each Member State, through a “give and take” process that involves Country Reports and the development of National Reform Programmes, to determine its specific challenges in achieving these goals. Based on these findings, the EC then issues country-specific recommendations (CSRs) in areas that require work, which have to be adopted by the Council of the EU (ECOFIN). Depending on the EU competences, these are either directly legally enforceable or intended as guidance to the Member States, supported by funds. 

Initially, the emphasis in the Semester process was on economic and fiscal sustainability, often at the expense of public expenditure reductions [64]. This is reflected in the Macroeconomic Imbalance Procedure (MIP), which was part of a series of other measures designed to identify and rectify economic imbalances in one Member State that had the potential to adversely impact others. The MIP set out economic indicators related to excessive deficit and debt and incorporated financial penalties for instances of persistent non-compliance [65]. The structural reforms proposed within the CSRs addressed aspects of Member States’ welfare systems, urging them to alleviate the strain on public finances through public expenditure cuts and an increase in taxation [66,67]. 

From an early stage, this one-sided approach of the European Semester to foster fiscal stability raised persistent doubts and criticism from social and public health actors regarding its compatibility with social objectives. Measures introduced through the European Semester resulted in the degradation of social services in many parts of the EU, especially in those countries most affected by the financial crisis in 2010 (notably, Portugal, Italy, Ireland, Greece and Spain), with often dire consequences for health and social outcomes. This ran counter to the provisions in the EU Treaties that stated that the EU would strive for “balanced growth”, in ways that protected and promoted public health and ensured social progress and wellbeing. The recommendations are regarded by many as contributing factors to the EU’s social crisis and a catalyst for the rise of national populism in the early 2010s [7].

### 3.4. Socialising the Semester Process under the Juncker Commission

The European Commission, led by Jean-Claude Juncker (2014–2019), as well as other European institutions recognised the need and political opportunity to strengthen the EU’s coordination of national social and employment policies, to promote a stronger “market-correcting” approach in the Semester [59].

The initial focus of the Juncker Commission in the realm of social policy was to promote the measures outlined in the EU “Social Investment Package” (2013), coordinated by DG Employment and Social Affairs (DG EMPL), that coupled measures in the field of education, skills and jobs, with social protection and stabilisation. In addition, DG EMPL was included by the Secretary General, as the driver of the European Semester process, to be a part of the “core” group of other Directorate-Generals (DGs) involved in the Semester, like the DG for Economic and Financial Affairs (DG ECFIN) and the DG for Internal Market, Industry, Entrepreneurship and SMEs (DG GROW). 

As a result of these measures, a growing number of CSRs addressed to the Member States in the 2015, 2016 and 2017 cycles increasingly included social, employment and educational policy topics [59]. For instance, while the total number of CSRs issued dropped from 157 in 2014 to just 89 in 2016, the share of recommendations related to employment, social, educational and tax policies incrementally rose from 47% in 2014 to 60.5% in 2017, although it was driven by a lower number of concrete recommendations [62].

An even more pronounced integration of the social dimension within the European Semester was the development and launch of the European Pillar of Social Rights (EPSR) in 2017. The EPSR establishes 20 principles that the EC considers essential for fair and well-functioning labour markets and welfare systems, with rights organised into three main areas: (1) equal opportunities and access to the labour market; (2) fair working conditions; (3) social protection and inclusion. The EPSR was accompanied by a Social Scoreboard, a set of indicators used to monitor the progress in the various principles and benchmark EU Member States’ performances on the EPSR. The fact that the Secretary General took the lead developing and promoting the EPSR, which the principles included as they were considered basic rights, and that it was adopted in a “tripartite agreement” with the European Parliament and the Council of the EU strengthened its profile across the EU. 

When introducing the EPSR, the EC indicated that it should be mainstreamed as a point of reference for a further implementation of the European Semester and for national governments to consider as part of their National Reform Programmes. Direct references to the EPSR were made across the European Semester documents in the 2018 Semester cycle. The Pillar and the Scoreboard served as new, concrete reference frameworks to justify further attention to social and employment issues in the Semester process.

These were amongst the significant steps taken by the Junker Commission to ensure that the European Semester process could lead to more balanced economic reform processes in EU Member States. The process remained quite siloed however, with different departments responsible for issuing different CSRs and little consideration for how they interacted. The overall emphasis, for example, remained on protecting and investing in growth/economic capital. The stronger focus on human and social capital served, above all, the economy, with less emphasis on how the economy itself might be compounding social inequities and undermining health and wellbeing. In this regard, many social actors including EuroHealthNet continued to call out the need for more coherence across the CSRs [68].

A Court of Auditors report (2020) on the Semester’s achievements in contributing to the Europe 2020 Strategy raised the lack of a strategic focus of the CSRs. It found that it was generally unclear how these contributed to the Annual Growth Survey and the Europe 2020 Strategy overall, as well as how they would be implemented and their progress evaluated. The report also mentioned a lack of consideration for how CSRs might interact with one another, be in conflict or be mutually dependent. There was also a lack of transparency as to why certain issues were prioritised over others and how they would be implemented and funded or not. In addition, the EU’s funding programmes lacked linkages to the priorities of the European Semester at the time [69]. The Court of Auditors report also set out how the Member States’ implementation of the CSRs has been modest and uneven and has worsened in recent years. Other authors, on the other hand, have pointed to the Semester process’ and the CSRs’ contribution to Member States’ “institutional capacity”, namely “the capacity to shape the outcomes of policy-making through expertise or political arguments, thus influencing deliberations on the drafting of reform advice or the interpretation of statistics” [60,61].

### 3.5. “An Economy That Works for People and the Planet” under the Von Der Leyen Commission

The Von der Leyen Commission (2019-2024) has built on the developments of the Junker Commission. From the outset, this Commission has faced one of the most tumultuous mandates in the history of the European Union, confronting an ongoing series of crises like the COVID-19 pandemic, the worsening climate crisis, the emergence of new global conflicts, the escalation of international instability, the high cost-of-living and aggravating concerns about migration.

While many of the policy priorities of the Von der Leyen Commission resemble those of its predecessor, it placed a much stronger emphasis on green and digital transitions. There was a shift in the narrative to emphasise “competitive sustainability” through an “*economy that works for the people and the planet*” and by “*upgrading the EU’s unique social market economy*” [70]. At the outset of its mandate, the Von der Leyen Commission presented the European Green Deal, which aims to make Europe the first climate-neutral continent, by 2050. The Green Deal was positioned as the EU’s new growth strategy and includes ambitious goals in the fight against climate change. To reach these targets, the EC created an Action Plan, which includes, as one of its key elements, the need to take everyone along, through a “just” and “fair” transition [71]. 

**Figure 2:**
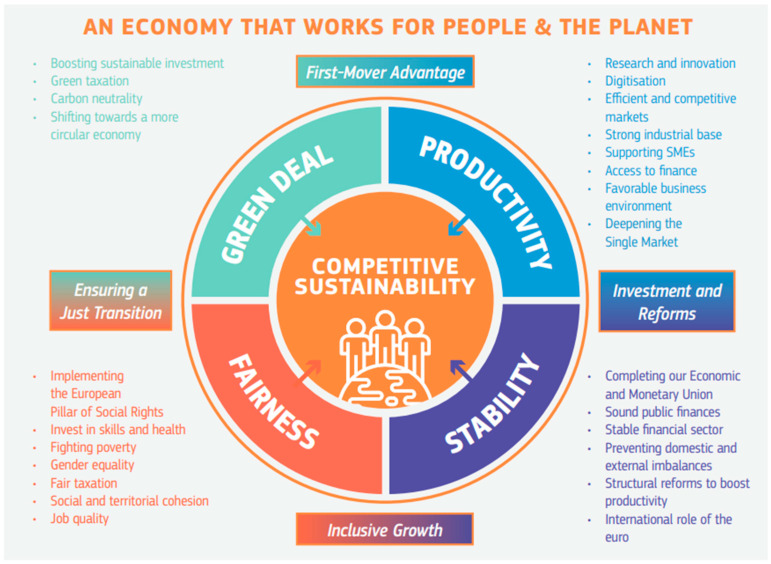
2020 European Semester: Annual Sustainable Growth Strategy [72].

To support the rhetoric, the Von der Leyen Commission significantly overhauled the European Semester as early as the 2020 cycle with the intention of elevating social and environmental concerns to a comparable level as financial stability and economic priorities. What began as the Annual Growth Survey, later re-named the Annual Sustainable Growth Survey or Strategy, was re-structured around four interconnected dimensions needed to achieve “competitive sustainability”, namely environmental sustainability, fairness, productivity gains and macroeconomic stability [72] (See Figure 2). This reflects a move to a more integrated policy-making style to overcome the traditional siloed approach and identify interlinkages between environmental, economic and social objectives [4].

The Von der Leyen Commission also strengthened the culture of preparedness and strategic foresight at the EU level and put forward resilience as a new compass for EU policies. This led to the development of “resilience dashboards” in four interrelated dimensions: social and economic, green, digital and geopolitical, to help EU Member States identify existing and emerging challenges to build stronger, more resilient societies [73]. In addition, the Commission indicated that they would put the UN SDG at the heart of EU policy. This was conducted by including them, as well as the resilience dashboards, into the European Semester process, through dedicated annexes setting out progress in the Country Reports, allowing for more effective monitoring [74].

The Von der Leyen Commission also announced and developed an ambitious Action Plan to enhance the implementation of the European Pillar of Social Rights, setting out three targets for 2030 relating to unemployment reduction (78% of people aged 20–64 in employment), skills (at least 60% of adults in training every year) and social inclusion (the number of people at risk of poverty and social exclusion reduced by at least 15 million). In the Social Pillar Action Plan, the EC reiterated its intention to employ the European Semester to offer guidance on the implementation of the EPSR principles at the national level, which includes utilising the relevant EU funding mechanisms [75]. The Social Scoreboard was also revised, to include an updated and expanded set of indicators to track progress towards the EPSR principles in a more comprehensive manner and to monitor the implementation of policy actions proposed by this Action Plan [4].

The Von der Leyen Commission’s commitment to a more “reform focused” agenda became apparent in its response to the COVID-19 pandemic. Instead of pushing for cuts in public spending as an approach to address the crisis, a process was initiated that led to the *NextGenerationEU* investment tool and its Recovery and Resilient Facility (RRF) to support Member States to overcome the impact of the pandemic whilst also addressing long-standing challenges. The EU Institutions activated the general escape clause of the Stability Growth Pact (2020–2023), effectively suspending the enforcement of the fiscal rules under the Macroeconomic Imbalance Procedure. This represented the first deviation from policy reforms aimed at achieving financial sustainability and macroeconomic stability since the launch of the European Semester process, a more relaxed approach towards budgetary matters and an emphasis on issues like the adequacy of national protection systems. The RRF was linked to the social objectives set out in the European Semester in 2019 and 2020, which, as a result of the COVID-19 pandemic, included a much stronger emphasis on strengthening health systems, ensuring the functioning of social security services and supporting the digital skilling of workers [76].

The Commission obliged Member States to fully implement the EPSR and Social Scoreboard and integrate these in the objectives of the national Recovery and Resilience Plans [59]. While the EC’s guidelines to Member States set out that 37% of expenditures must go to investments and reforms that support the ecological transition/climate objectives and 20% must foster the digital transition, they did not, however, include guidelines around the percentage of the funds to invest in social priorities.

Von der Leyen’s Commission’s rhetoric around “an economy that works for people and the planet” is reflective of a Wellbeing Economy, since it puts the emphasis on how the economy is serving people, rather than on how people can serve the economy. The European Semester process, which began as a tool that focused exclusively on fiscal and economic policy coordination, has been broadened to incorporate social and environmental considerations, as reflected by the new structure of the Annual Sustainable Growth Strategy. The reports produced by the EU Institutions and EU Member States now reflect on the economic performance of EU Member States from a much wider range of dimensions [62]. It remains unclear, however, how the wealth of information gathered in each of the Country Reports is weighed up against each other and the annual strategic focus of the Semester process, to result in the CSRs received by each country. Many of the Court of Auditors’ recommendations from their 2020 report [69], regarding the focus of the CSRs and how they interact with one another, remain relevant. Given the EU’s institutional emphasis on fiscal and economic considerations and “growth” as well as its stronger governance capacities to scrutinise performance in these domains, there is danger that social and environmental concerns are being subordinated to fiscal and macroeconomic objectives, especially now that the EU’s fiscal rules that were temporarily suspended during the COVID-19 crises have been reinstituted. The final part of this article will elaborate on this and the measures needed to further strengthen the European Semester as a governance mechanism to achieve economies that generate more wellbeing, in the EU.

## 4. Reforming the European Semester to Shape Economies of Wellbeing

As set out at the outset of this paper, the concept of Economies of Wellbeing emerged from growing awareness that economic growth is not an end in itself. The pursuit of GDP growth is in many ways undermining the very things that it should ultimately contribute to, namely, as set out in the Treaties of the EU, peace, our values and wellbeing. Central to the concept of Wellbeing Economies is the need to shift the focus from growth, to ensure economic activity or growth is in the service of peoples’ wellbeing. Achieving Economies of Wellbeing therefore calls for societies to define what matters the most to ensure humanity survives and thrives, to establish clear policy priorities that relate to this and to bring these policy priorities together in a clear, comprehensive, coherent and integrated strategy, where all sectors are encouraged and enabled to share responsibility for all priorities, rather than just their own. Achieving this more cross-sectoral approach calls for the right governance mechanisms and tools to enable all sectors to deliver on these joint priorities as efficiently and effectively as possible, by identifying synergies and addressing tensions and trade-offs. 

The Finnish EU Council Presidency of the EU issued Conclusions invited the EU and Member States to engage in the process of achieving Economies of Wellbeing. They called on them “to develop a Strategy to make the EU the most competitive, inclusive and environmentally sustainable economy”. The Council Conclusions also state that the Union should “… adopt an ambitious framework to “undertake a horizontal analyses within the European Semester process in order to enhance broad, long-term policy perspectives and provide balanced policy recommendations” [30].

As set out in Part 2, the European Union has in many ways heeded this call, by reforming the Annual Sustainable Growth Strategy. The Strategy integrates the EU’s priorities in relation to environmental and social policy while ensuring stable and productive economies. It provides a framework for coordinating the economic and employment policies of the EU Member States, in ways that align with the UN SDGS and take into account the outcomes of the new resilience dashboards. The country reports that emerge from the process provide a rich and more holistic analysis of developments as well as challenges that EU Member economies are facing. This information is used to encourage or compel Member States, depending on the levers that the EU has available, to reform their economies to ensure they have the flexibility to tackle existing challenges and achieve short-term goals, in ways that align with long-term objectives. The CSRs set out how each Member State can reform their economies, in ways that align with the EU’s annual strategic priorities. The European Commission’s 2023 Spring Package Communication notes that horizontal coordination in the process has improved and that the country reports and CSRs are being developed on the basis of strengthened dialogue processes that take place during all stages of the Semester process, between EU institutions and social partners and stakeholders at the national and European level [77]. 

Upon further consideration however, the ASGS falls considerably short of what could be considered a “Wellbeing Strategy”, since its objectives are ill defined, and it is not comprehensive enough for this. In addition, because the main goal is “growth”, the language and perspective taken reflect that economic and fiscal considerations predominate over other considerations, impeding the potential true integration and cross-sectoral collaboration in relation to policy priorities. Regarding the first point, the objectives of the ASGS, namely, to achieve “competitive sustainability” through “economies that work for people and planet”, are ill defined. The level of detail in the National Country Reports and the fact that the indicators analysed in the context of the four dimensions of the ASGS are not linked to any overarching objectives in each of the four areas make it difficult to obtain a clear picture of whether and how Member States are progressing in each dimension. It also makes it difficult to compare Member States’ performance and to assess the EU’s overall progress in achieving economies that work for people and the planet. In addition, the ASGS’s strategic priorities differ from year to year, depending on trends identified and set out in the Annual Sustainable Growth Survey. In 2023, for example, the strategic priority was to mitigate the impacts of the energy shocks, while the priority for 2024 is to implement the RRPs and complement this with other funding instruments, like Cohesion Policy [78,79]. Given the lack of more specifically defined overarching objectives, targets and indicators, there is no way to determine if the measures taken by Member States to comply with the recommendations are effective or not, in contributing to the overall goals of the ASGS. 

In addition, although the ASGS has been broadened to include social and green priorities, it lacks consistency and coherence as a Wellbeing Strategy, while the language used to describe the different dimensions of the Strategy is not commensurate to wellbeing. From a wellbeing perspective, the overarching goal of “sustainable competitiveness” can be considered a contradiction in terms. While competition can be healthy in a marketplace, to ensure a fair playing field and avoid monopolies, it also suggests winners and losers and is at odds with concepts of connection, integration, regeneration and redistribution, which are more aligned with notions of inclusiveness, sustainability and wellbeing. As set out in the Annual Sustainable Growth Survey, competitiveness alludes to the hostile geopolitical environment that marks our times due, for example, to the wars in Ukraine and Gaza and tensions with China [79]. The concept of competitiveness in the ASGS therefore refers to the need for physical and material security and to maintain peace and the European values of democracy, freedom and justice. Viewed together in this way, the dimensions included in the ASGS are reflective of the Strategic Priorities set out in the European Commission’s Strategic Agenda 2019–2024 [80]. These priorities also overlap with those identified during the Conference on the Future of Europe that took place between April 2021 and May 2022 and enabled Europeans to share their views around this theme [81]. All of these priorities, however, fail to include some important dimensions of what matters the most to people that are included in the OECD and Doughnut Economy model and that have been identified by WEGo countries, like those relating to health, work/life balance, social connections, knowledge and skills. If the ASGS were to be considered a “Wellbeing Strategy” like the one recommended in the Finnish EU Council Conclusions, its priorities would need to be better articulated to inspire and engage and expanded, to include some more dimensions of what matters to people, while these priorities would need to be presented in a more balanced manner. 

This links to the final reason that the Annual Sustainable Growth Strategy underpinning the European Semester process cannot be considered a Wellbeing Strategy: economic and fiscal considerations continue to prevail over environmental or social considerations, while the latter are equally important to wellbeing. The main objective of the ASGS is growth, albeit “green and inclusive” growth, following the tenets of the upgraded “social market economy model”. This means that economic dimension is implicitly prioritised over the other dimensions that are included or alluded to. This bias is apparent from the language used and the perspective taken in the documents setting out the annual priorities of the Semester process and of the CSR’s. The Annual Sustainable Growth Survey for 2024, for example, stresses the need to “increase labour market participation to improve employment and social outcomes” [79]. It could also have taken a different entry point and focused on the need to improve social outcomes, to improve wellbeing and thereby increase participation and productivity. Similarly, the report setting out CSRs for Belgium [82] notes the need to pursue policies that boost the reintegration of workers on long-term sick leave, in particular due to burn out and depression (25% of the cases) who now largely exceed the number of unemployed, to address skills shortages and reduce the unemployment rate. It does not focus on or consider the issue from the perspective of why workers burned out and became depressed in the first place, which can result from an overemphasis on productivity or the lack of a sense of meaning or purpose in one’s work. In other words, despite the rhetoric of an economy that works for people and the planet, the focus remains on how people and the planet can contribute to the economy, to thereby generate the wealth needed to achieve the EU’s social, environment and digital objectives. While this approach has in many ways generated prosperity in Europe, it has also, as set out in Part 1, led to many inefficiencies and market failures that undermine wellbeing and require additional public spending to “fix” this. 

The prioritisation of economic considerations is also evident in the RRF guidelines that have been integrated into the Semester guidelines. While the guidelines include targets for Member States’ RRF spending on green and digital priorities, this is not the case for social priorities, where the EU simply encourages Member States to invest funds, in ways that contribute to the implementation of the European Pillar of Social Rights. It has also been noted that the EC’s RRF guidelines hardly make reference to the UN SDGs, which reflects a lost opportunity by the EU to encourage Member States to take further action in areas where data reflect that they are not doing well [83].

The economic and fiscal bias in the ASGS makes sense, given the European Semester began as a process to coordinate Member States’ economic and fiscal policies and is managed by economic and fiscal experts. The language and the perspective taken nevertheless reinforce that the ASGS remains, to a large extent, an economic and employment strategy, which to a large degree, overlaps with the EC’s new “Competitiveness” Strategy [84]. While the need to ensure macroeconomic and fiscal stability, employment opportunities and productivity is important, they should not be considered ends in themselves, even if the jobs are “green” and “fair”. The measure of success, as indicated in the TEU, should be whether or not these dimensions ultimately contribute to wellbeing and ensure people can meet their material needs, feel safe, connected and that they can contribute and matter.

A true Wellbeing Strategy would therefore make wellbeing the central goal and consider all wellbeing priorities in a more balanced way and would generate mechanisms, tools and metrics to encourage discussion that focused on the question of different sectors’ roles in contributing to the different priorities and where synergies could be made, what tensions are apparent and what trade-offs are necessary, as discussed in Section 2.4. “Growth” could still be an objective but one achieved in the process of working towards all objectives, rather than the central one. 

It will be enormously challenging to shift from growth to wellbeing as a measure of success and to develop the tools and mechanisms needed to act on and effectuate this change. Amongst the main factors impeding this shift is the deep-rooted nature of the “growth mindset” within our societies, the vested interests of people and organisations that benefit from the status quo. It will also be difficult to obtain traction in efforts to create a new mindset, in societies where views are increasingly polarised. Another challenge lies in designing and implementing policies, tools and mechanisms that enhance wellbeing not just within but also beyond one’s borders and in addressing vested interests in that respect, too. At stake, however, may be the very survival of people, on this planet. Conversely, making a shift to wellbeing as a measure of success can lead to new synergies and approaches that enhance the wellbeing of many.

**Figure 3:**
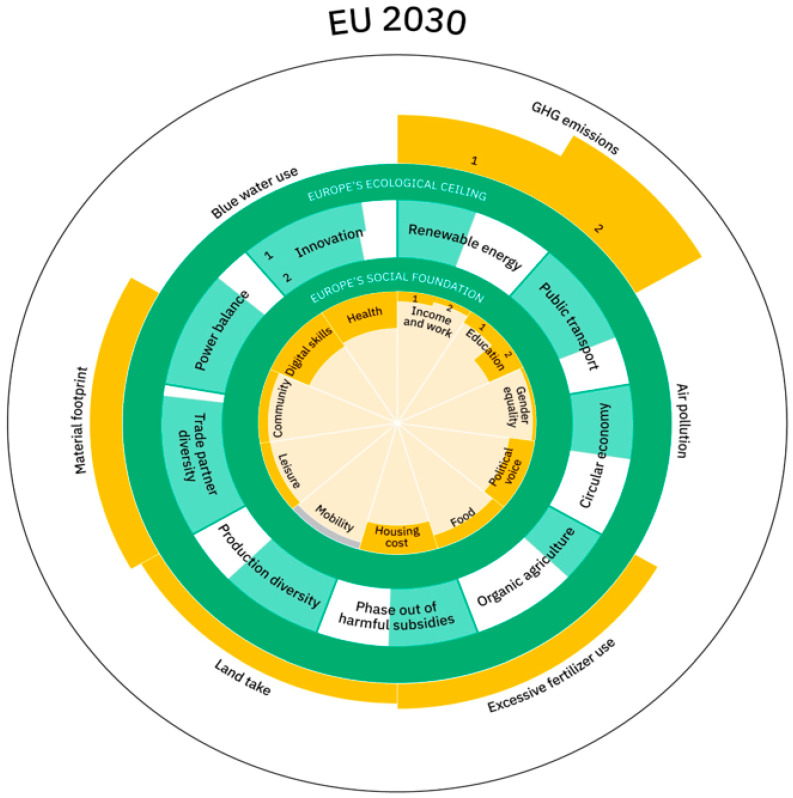
The EU 2030 Portrait [20].

One way to overcome this formidable challenge of achieving a change in mindset is to develop a clear strategy and accompanying framework that communicates well and helps all stakeholders visualise what a Wellbeing Economy entails and the progress that is needed. An inspiring example is the EU Doughnut by 2030 (see Figure 3), which presents a political summary of existing dashboards and selected 30 indicators that help the public understand what is required to transform economic systems while making policy design processes more effective [20,83].

The European Semester process is the main EU governance tool that is being used to harmonise and coordinate policies across EU Member States to, ultimately, achieve “an economy that works for people and planet” and wellbeing. While the process is therefore of significant relevance to a very wide range of actors at all policy levels across Europe, many disengage, considering it too economic, technocratic and irrelevant to them. Putting the process at the service of a broader Wellbeing Strategy, underpinned by such a clear framework like the EU Doughnut by 2030, will make it easier for different stakeholders to see its relevance and more enticing to engage.

Today’s grave geopolitical challenges may reinforce the belief that we must above all focus on economic growth and competition to maintain peace and security. Peace, security and sound economies are essential to wellbeing. Yet, becoming more competitive or striving for security in ways that continue to erode other key elements of wellbeing, like the environment, health, social connection and equity, and that build economic capital, at the expense of environmental, social and human capital, will ultimately weaken the EU. All policies are interrelated, and geopolitical conflicts and tensions, environmental crises and the digital transition cannot be seen in isolation of one another. Ineffective or underinvestments in the social arena (social protection, education, skills), for example, give political actors who do not share the values of democracy political leverage and economic actors the ability to make profits, at the expense of individual and societal wellbeing. Conversely, supporting wellbeing through greater investments in human and social capital is crucial for cultivating respect for peace and the values of democracy and human rights and for contributing to building and ensuring the resilience and capacities that people need to defend these and their interests. It is therefore important to develop a framework and strategy that bring these factors together, in a balanced way. This can expose better the synergies that can be achieved, tensions to be addressed and the trade-offs that must be negotiated and addressed, across policy levels. 

The following recommendations set out what can be conducted to strengthen the European Semester process to achieve economies that deliver greater wellbeing:

1. Work in coalitions to establish how to best communicate the concept of Wellbeing Economies and the need to move from a “growth” to a “wellbeing” mindset and promote this message widely, within the public sector and beyond. Stimulate the interest and engagement of all relevant stakeholders in the Semester process by framing it as a potential “Wellbeing Economy” process.
2. Work in coalitions, towards the development of a coherent, consistent overarching EU-level Wellbeing Strategy, to demonstrate to politicians and policy-makers the potential of such a Strategy. Identify Wellbeing priorities that are the most relevant to the EU level, drawing from, e.g., the EU’s Strategic Agenda priorities, the outcomes of the Future of Europe process and the results of other EU-wide surveys like the EU Social Survey. Establish potential targets and indicators that can be used to reflect these priorities, to develop a “wellbeing dashboard”. Such a dashboard and mechanism can make it easier to understand the progress being made across EU-level policy objectives, to steer EU-level funding, identify where policy initiatives can reinforce one another and what trade-offs need to be made. The ZOE Institute’s “Beyond GDP” dashboard is an inspiring example of what can be conducted [20].
3. In the short term to medium term, pursue other initiatives that can lead to a more balanced consideration of different policy areas, within the European Semester process. The Employment, Social Policy, Health and Consumer Affairs (EPSCO) Council is becoming more involved in the Semester process, discussing the need for a Social Convergence Framework to better integrate the EPSR in the process [85]. These discussions build on calls by the Belgian and Spanish governments, for a Social Imbalance Procedure to match the Macroeconomic Imbalance Procedure [86]. Many organisations have called for a “golden rule”, to exclude green, health and social investments from EU national debt rules [62,87]. These initiatives can, over the longer term, be integrated into and contribute to the development of a wellbeing dashboard and a wellbeing alert mechanism. Many social, health and environmental bodies are also calling for an EU Parliamentary Group on Beyond GDP that can engage in and contribute to this process.
4. Appoint a Commission Vice President for a Wellbeing Economy that is responsible for ensuring the coherence and consistency of policies at the EU level and their integration and coordination, to ensure initiatives reinforce one to achieve the EU’s strategic (wellbeing) priorities and objectives. The Vice President will also be responsible for identifying and making trade-offs more explicit and managing their resolution through democratic negotiation. The Commission Vice President for a Wellbeing Economy will ultimately be responsible for the design, implementation and evaluation of the overarching EU Wellbeing Economy framework and Strategy, which structures the European Semester process and ensures all dimensions are considered equally, for more efficient and effective governance processes, to achieve the European Union’s overarching aims of promoting peace, its values and wellbeing.

## 5. Conclusions

This paper has set out what a Wellbeing Economy means and how it can be achieved, in the context of the EU and the European Semester process. There is still a long way to go, to shift the focus beyond the growth of products and services, towards the growth of societal wellbeing. Significant progress can be made by putting the European Semester process at the service of an overarching Wellbeing Strategy and a clear framework, which brings together key EU-level policy priorities and objectives in a more balanced way. Progress can also be made through the development of tools, governance mechanisms and metrics that go “beyond” GDP to emphasise in equal measure other aspects of wellbeing. An overarching EU-level Strategy, underpinned by such governance tools and metrics, would help to ensure more coherent, consistent and integrated policies and facilitate cross-sectoral collaboration towards wellbeing, as the main objective of public policy. 

Tackling climate change, maintaining the biodiversity of our planet and ensuring the production of resources that are fairly distributed to protect and promote everyone’s health and wellbeing while maintaining peace and the values of justice, freedom and democracy are all equally urgent priorities. These can be addressed through the recommendations set out above. More work is also needed to analyse and expose imbalances across policy priorities and to determine where synergies can be made, tensions addressed and where trade-offs are required across areas, to protect and promote wellbeing. EuroHealthNet will continue to work across sectors, bridging policy, research and practice, to contribute to this agenda.

## Figures and Tables

**Table 1:** Terminology relevant to ensuring more horizontal, integrated policy approaches.

**Policy integration** refers to holistic thinking beyond specific policy areas within policy design processes. **Policy coherence** refers to holistic approaches and the extent to which various policies work well together and whether there are (missed) opportunities, possibilities or synergies between different policy areas. **Policy consistency** refers to whether there are inconsistencies or contradictions between individual elements of policies that impact the effectiveness of policies. **Policy coordination** refers to harmonising tasks, efforts and understanding between different government sectors and agencies, across governance levels. **Trade-offs** exist if conflicts between policy areas are deterministic and an improvement in one objective will lead to a deterioration in another objective. In that case, there must be a prioritisation of policy objectives. **Tensions** exist if the improvement in one objective can lead to deterioration but does so only depending on the contexts that may be modifiable.

## Data Availability

No new data were created or analyzed in this study. Data sharing is not applicable to this article.
